# Protective effects of N-acetylcysteine on acetic acid-induced colitis in a porcine model

**DOI:** 10.1186/1471-230X-13-133

**Published:** 2013-08-30

**Authors:** Qingjing Wang, Yongqing Hou, Dan Yi, Lei Wang, Binying Ding, Xing Chen, Minhui Long, Yulan Liu, Guoyao Wu

**Affiliations:** 1 Hubei Key Laboratory of Animal Nutrition and Feed Science, Wuhan Polytechnic University, Wuhan 430023, China; 2 Department of Animal Science, Texas A&M University, College Station, TX 77843, USA; 3 State Key Laboratory of Animal Nutrition, China Agricultural University, Beijing 100193, China

**Keywords:** N-acetylcysteine, Acetic acid, Colon injury, Claudin-1, Epidermal growth factor, Amphiregulin

## Abstract

**Background:**

Ulcerative colitis is a chronic inflammatory disease and involves multiple etiological factors. Acetic acid (AA)-induced colitis is a reproducible and simple model, sharing many characteristics with human colitis. N-acetylcysteine (NAC) has been widely used as an antioxidant in vivo and in vitro. NAC can affect several signaling pathways involving in apoptosis, angiogenesis, cell growth and arrest, redox-regulated gene expression, and inflammatory response. Therefore, NAC may not only protect against the direct injurious effects of oxidants, but also beneficially alter inflammatory events in colitis. This study was conducted to investigate whether NAC could alleviate the AA-induced colitis in a porcine model.

**Methods:**

Weaned piglets were used to investigate the effects of NAC on AA-induced colitis. Severity of colitis was evaluated by colon histomorphology measurements, histopathology scores, tissue myeloperoxidase activity, as well as concentrations of malondialdehyde and pro-inflammatory mediators in the plasma and colon. The protective role of NAC was assessed by measurements of antioxidant status, growth modulator, cell apoptosis, and tight junction proteins. Abundances of caspase-3 and claudin-1 proteins in colonic mucosae were determined by the Western blot method. Epidermal growth factor receptor, amphiregulin, tumor necrosis factor-alpha (TNF-α), and toll-like receptor 4 (TLR4) mRNA levels in colonic mucosae were quantified using the real-time fluorescent quantitative PCR.

**Results:**

Compared with the control group, AA treatment increased (*P* < 0.05) the histopathology scores, intraepithelial lymphocyte (IEL) numbers and density in the colon, myeloperoxidase activity, the concentrations of malondialdehyde and pro-inflammatory mediators in the plasma and colon, while reducing (*P* < 0.05) goblet cell numbers and the protein/DNA ratio in the colonic mucosa. These adverse effects of AA were partially ameliorated (*P* < 0.05) by dietary supplementation with NAC. In addition, NAC prevented the AA-induced increase in caspase-3 protein, while stimulating claudin-1 protein expression in the colonic mucosa. Moreover, NAC enhanced mRNA levels for epidermal growth factor and amphiregulin in the colonic mucosa.

**Conclusion:**

Dietary supplementation with NAC can alleviate AA-induced colitis in a porcine model through regulating anti-oxidative responses, cell apoptosis, and EGF gene expression.

## Background

N-acetylcysteine (NAC), the precursor of L-cysteine and therefore reduced glutathione, has been widely used as an antioxidant in vivo and in vitro [1]. NAC is rapidly metabolized by the small intestine to produce glutathione [2] and is usually not detectable in the plasma or tissues of pigs receiving no NAC supplementation [3]. Previous studies have shown the protective effect of NAC against the toxicity of chemicals due to its dual role as a nucleophile and as a -SH donor [4]. Specifically, NAC acts as a direct ROS scavenger to regulate the redox status and also affects several signaling pathways involved in apoptosis, angiogenesis, cell growth and arrest, redox-regulated gene expression, and inflammatory response [5,6]. Moreover, NAC exerts an indirect antioxidant effect through the synthesis of glutathione, a primary intracellular factor against toxic agents [7]. Therefore, NAC may not only protect against the direct injurious effects of oxidants, but also beneficially alter inflammatory events [8].

Inflammatory bowel diseases (IBD), including Crohn’s disease (CD) and ulcerative colitis (UC), are complex disorders characterized by chronic, local, and systemic inflammation as well as a spontaneously relapsing course [9]. Ulcerative colitis is a chronic inflammatory disease [10] and involves multiple etiological factors [11,12]. A large body of evidence suggests that oxidant derivatives and reactive oxygen species (ROS) are produced in excess by the inflamed mucosa and may be pathogenic factors in IBD [13,14]. Oxidative stress have an important bearing on inflammation via the activation of redox-sensitive transcriptional factors such as nuclear factor kB (NF-kB) and activator protein 1, which regulate expression of key genes encoding pro-inflammatory mediators and protective antioxidant proteins. In support of this view, pharmacological agents that lower the amounts of reactive oxygen metabolites may reduce inflammation [13]. Many animal models of IBD have been developed to study its pathogenesis and therapeutic means [15]. Acetic acid (AA)-induced colitis is a reproducible and simple model, sharing many characteristics with human colitis [11,16].

In the intestinal tract, energy status is a fundamental regulator of epithelial cell metabolism [3]. An energy deficit has been considered to be a pathogenic factor in ulcerative colitis, which is substantiated by the fact that the intestinal mucosa has a limited capacity for *de novo* synthesis of purine nucleotides [17] and is more prone to reduced ATP concentrations compared with the liver or muscle [18]. On the other hand, IL-6 and tumor necrosis factor α (TNF-α) has been shown to play an important role in the pathogenesis of inflammatory bowel disease [19]. These pro-inflammatory cytokines drive the activation and recruitment of inflammatory cells, amplify the production of other pro-inflammatory cytokines, and activate nuclear transcription factors, thereby promoting and maintaining the inflammatory response [20]. Additionally, release of transforming growth factor-α (TGF-α) and expression of TGF-α mRNA are increased after acute gastric injury and in the colonic mucosa from patients with IBD [21,22].

Neonates are prone to various stresses, such as early-weaning, inflammatory bowel disease, and infection, resulting in intestinal mucosal injury and absorptive dysfunction [23-25]. However, effective prevention and treatments are currently limited [26]. Many nutrients (vitamin E, selenium and trimetazidine) have been investigated as possible agents to protect animals against the IBD. Dietary supplementation with vitamin E and selenium reduced both the severity of colonic lesions and the levels of malondialdehyde (MDA) [27,28]. Likewise, intraperitoneal administration of trimetazidine improved macroscopic and microscopic scores and decreased colonic myeloperoxidase (MPO) activity in rats receiving administration of AA [29]. In previous studies, we have reported that NAC reduced inflammation, alleviated oxidative stress, improved energy status, and ameliorate tissue damage in the small intestine of piglets [2,30]. Thus, we postulated that dietary supplementation with NAC may alleviate the AA-induced colonic injury in piglets. The purpose of the present study was to test this hypothesis and to elucidate the underlying molecular mechanisms. As the piglet is a well-established animal model for studying human gastrointestinal disease, findings of this study will provide vital clues for prevention of human colitis.

## Methods

### Animal care and diets

The animal use protocol for this research was approved by the Animal Care and Use Committee of Hubei Province. Eighteen healthy crossbred female piglets (Duroc × Landrace × Yorkshire), which were reared by sows, were weaned at 21 days of age. After a 7-day period of adaptation, piglets (average body weight of 6.44 ± 0.39 kg) were housed individually in stainless steel metabolic cages (1.20 × 1.10 m^2^) and maintained in an environmentally controlled room (25°C) by air conditioning, with electric light being provided between 8:00 AM and 8:00 PM [26]. Each cage was equipped with a feeder and a nipple waterer to allow piglets free access to food and drinking water [26,31-33]. The corn- and soybean meal-based diet was formulated to meet National Research Council (NRC 1998) requirements for all nutrients [2].

### Experimental design

In the first week, all weanling piglets had free access to the basal diet to help them adapt to solid food. Then, eighteen healthy piglets were allocated randomly into one of the three treatments: 1) control group (piglets fed the basal diet and receiving intrarectal administration of 10 mL of sterile saline); 2) AA group (piglets fed the basal diet and receiving intrarectal administration of 10 mL of 10% AA); 3) NAC group (piglets fed the basal diet supplemented with 500 mg/kg NAC and receiving intrarectal administration of 10 mL of 10% AA). NAC (powder) was well mixed with the basal diet. Diets for the control and AA groups were supplemented with 500 mg/kg cornstarch to obtain approximately isocaloric diets. The dosage of NAC was chosen according to the results of our previous study indicating that dietary supplementation with 500 mg/kg NAC could ameliorate growth depression and restore intestinal function in weanling piglets [2,30]. It is unnecessary to use non-essential amino acids as an isonitrogenous control because the dietary supplementation with 500 mg/kg NAC only resulted in an increase of 0.0042% nitrogen. On day 15 of the trial, piglets in the AA and NAC groups received intrarectal administration of 10 mL of 10% AA, whereas the control group piglets received the same volume of saline. The dosage of AA was chosen according to the studies of Jurjus et al. [15]. During days 0–15 of the trial (pre-challenge), all the piglets had free access to feed and drinking water. To exclude a possible effect of AA-induced reduction in food intake on the piglet intestine, the control and NAC piglets were pair-fed the same amount of feed per kg body weight as AA piglets during days 15–21 of the trial (post-challenge with AA). On day 22 of the trial, all piglets were sacrificed by injection of sodium pentobarbital (50 mg/kg BW) to obtain the colonic mucosa for the evaluation of intestinal morphology and biochemical analysis [34].

### Blood sample collection

On day 22 of the trial, blood samples were collected from the anterior vena cava into heparinized vacuum tubes (Becton Dickinson Vacutainer System, Franklin Lake, NJ, USA), as we previously described [26]. Blood samples were centrifuged at 3,000 rpm for 10 min at 4°C to obtain plasma [26,35]. Plasma was stored at −80°C until analysis.

### Intestinal sample collection

The piglet abdomen was surgically opened immediately from the sternum to the pubis, and then the whole gastrointestinal tract was immediately exposed [26,36]. The large intestine, dissected free of the mesentery, was placed on a chilled stainless steel tray. Colon segments (5- to 10-cm) were obtained, flushed gently with ice-cold phosphate buffered saline (PBS, pH 7.4), and placed in 10% fresh chilled formalin solution for histological measurements [26,34]. Additional colon segments were opened longitudinally and the contents were flushed with ice-cold PBS [26,37]. Thereafter, the mucosa was collected by scraping using a sterile glass microscope slide at 4°C [26,38], rapidly frozen in liquid nitrogen, and stored at −80°C until analysis. All samples were collected within 15 min after sacrifice.

### Histologic assessments of colonic damage

Tissue samples for the morphometric study were dehydrated and embedded in paraffin, sectioned at 4 μm, and stained with hematoxylin and eosin [26]. Stained sections were determined for evidence of colonic injury using the following criteria: crypt lesion, bowel wall thickening, lymphocyte infiltration, goblet cell depletion, and denuded epithelium [9,39,40]. The degree of damage on microscopic cross-sections of the colon was graded semi-quantitatively using a score of 0 to 20. For example, grades of crypt lesion from 0 to 4 were as follows: 0: intact crypt, 1: loss of the one-third tissue, 2: loss of the two-third tissue, 3: loss of the entire crypt, 4: erosion [41]. The total possible score was 20 (absence of any abnormality = 0 and most severe injury = 20) [13]. Morphometric measurements were performed with a light microscope (American Optical Co., Scientific Instrument Div., Buffalo, NY, USA). Crypt depth (the distance from the crypt mouth to the base) was measured using a linear ocular micrometer with a computer-assisted morphometric system (BioScan Optimetric, BioScan Inc., Edmonds, WA, USA). Colonic intraepithelial lymphocyte (IEL) number and goblet cell number in crypts were measured. The variables were expressed per 100 enterocytes. Measurements were taken in 10 well-oriented crypts from each intestinal section of a study animal. On the basis of the cellular morphology, differences among the nuclei of enterocytes, goblet cells, and lymphocytes were clearly distinguishable at 400× magnification. Intra-villus lamina propria cell density was determined by counting total visibly stained nuclei and total lymphocytes in 8 fields from each section using a grid ocular (Olympus, Microplanet). Cell density was expressed as the number of total stained cells or the number of lymphocytes per 1,000 μm^2^[34]. The number of lymphocytes in relation to the number of total cells was also calculated. All morphometric analysis was done by the same person, who was blinded to the treatments.

### Measurement of mucosal DNA, RNA, and protein

DNA, RNA, and protein were extracted from the colonic mucosa, using TRI REAGENT-RNA/DNA/Protein isolation reagent and their concentrations were determined colorimetrically, as previously described [26]. Mucosal DNA was analyzed fluorimetrically using the method of Prasad et al. [42]. RNA was determined by spectrophotometry using a modified Schmidt-Tannhauser method as described by Munro and Fleck [43]. Protein was analyzed according to the method of Lowry et al. [44]. For measurement of colonic DNA and RNA levels, the mucosa was homogenized (~2 min) in a 100-fold volume of ice-cold saline (0.9%) and the homogenate was centrifuged at 1,800 × *g* for 10 min at 4°C to obtain the supernatant fluid for analysis. For measurement of mucosal protein, intestinal mucosal samples (~0.1 g) were homogenized using a tissue homogenizer in 1 mL of ice-cold PBS-EDTA buffer (0.05 mol/L Na_3_PO_4_, 2.0 mol/L NaCl, 2 mmol/L EDTA, pH 7.4) and the homogenates were centrifuged at 12,000 × *g* for 10 min at 4°C to obtain the supernatant fluid for assays.

### Assessments of antioxidant status

The colonic mucosa (~200 mg), homogenized in a nine-fold volume of freezing saline, was centrifuged at 2,500 rpm for 10 min at 4°C to obtain the supernatant fluid used for assays. Myeloperoxidase, superoxide dismutase (SOD), catalase (CAT), and malondialdehyde (MDA) in the plasma and colonic mucosa were determined using commercially available kits (Nanjing Jiancheng Bioengineering Institute, Nanjing, China).

### Determination of proinflammatory cytokines in the plasma and colonic mucosae

Frozen intestinal mucosal samples were powdered under liquid nitrogen, then homogenized in ice-cold 0.9% NaCl solution using a homogenizer (1 g sample/9 mL of 0.9% NaCl). The homogenates were centrifuged at 3,000 rpm for 15 min at 4°C to obtain the supernatant fluid [32].

Tumor necrosis factor-α (TNF-α) in plasma was analyzed using commercially available ^125^I RIA kits (Beijing North Institute of Biological Technology, Beijing, China). The detection limit was 0.3 ng/mL and the intra-and inter-assay coefficients of variation were 5% and 8%, respectively.

Interleukin-6 (IL-6) and prostaglandin E_2_ (PGE_2_) in plasma and the supernatant fluid of colonic mucosae were analyzed using commercially available ^125^I RIA kits (Beijing Sino-UK institute of Biological Technology, Beijing, China). The detection limits for interleukin-6 and prostaglandin E_2_ analyses were 50 and 0.12 pg/mL, respectively. The coefficients of variation for intra-and inter-assays of interleukin-6 were < 7% and < 15%, respectively. The coefficients of variation for intra-and inter-assays of prostaglandin E_2_ were < 7.5% and < 10.5%, respectively.

### Determination of EGF in plasma and TGF-α in colonic mucosae

Epidermal growth factor (EGF) in plasma and transforming growth factor-α (TGF-α) in colonic mucosae were analyzed using commercially available ^125^I RIA kit (Beijing Sino-UK Institute of Biological Technology, Beijing, China). The coefficients of variation for intra-and inter-assay of EGF were < 5% and < 10%, respectively. The coefficients of variation for intra-and inter-assay of TGF-α were 4.4% and 7.4%, respectively. The detection limit for EGF and TGF-α were 0.1 ng/mL and < 5 pg/mL, respectively.

### Protein immunoblot analysis

Analysis of caspase-3 and claudin-1 proteins in colonic mucosae were performed by western blot as described by Hou et al. [26]. Briefly, frozen samples were powdered under liquid nitrogen and homogenized in lysis buffer. The homogenates were centrifuged at 12,000 × g for 15 min at 4°C to get the supernatant fluid. A portion of this fluid is mixed with 2 × SDS sample buffer in a 1:1 ratio. The samples were boiled for 5 min and cooled on ice before western blot analysis. The proteins (60 μg/sample for caspase-3, claudin-1 and β-actin) were separated by electrophoresis on a 10% (for caspase-3) or 12% (for claudin-1) polyacrylamide gel. Proteins were electrophoretically transferred to a polyvinylidene difluoride (PVDF) membrane. Non-fat dry milk in TBS-T (1 × Tris-buffered saline including 0.1% Tween 20) was used to block membranes for at least 1 h at room temperature [26]. Membranes were then incubated with primary antibodies overnight at 4°C: caspase-3 (rabbit polyclonal antibodies from Cell Signaling Technology, Inc., Danvers, MA, USA; dilution 1:1000), claudin-1 (rabbit monoclonal antibodies from Invitrogen Technology, Inc., Danvers, MA, USA; dilution 1:1000), β-actin (mouse monoclonal antibody from Sigma Chemicals; dilution 1:5000). The membranes were washed three times for 10 min with TBS-T and incubated for 1 h at room temperature with anti-rabbit (mouse) immunoglobulin G horseradish peroxidase-conjugated secondary antibody (Beijing ZhongShan Golden Bridge Biological Technology Co., LTD, China; dilution 1:10000) [31]. Incubation of the secondary antibodies was followed by five washes for 8 min. Blots were developed using an Enhanced Chemiluminescence western blotting kit (ECL-plus, Amersham Biosciences, Sweden), visualized and quantified using an imaging system (Alpha Innotech FluorChem FC2, CA, USA) [2,30].

### Determination of EGFR, AR, TNF-α and TLR4 mRNA levels using quantitative real-time polymerase-chain reaction (RT-PCR)

Epidermal growth factor receptor (EGFR), Amphiregulin (AR), tumor necrosis factor-alpha (TNF-α) and toll-like receptor 4 (TLR4) mRNA levels in colonic mucosae were quantified using quantitative RT-PCR as described by Hou et al. [30]. Approximately 100 mg of a frozen mucosal sample, powdered under liquid nitrogen using a mortar and pestle, were homogenized in a buffer and total RNA was isolated using the TRIzol Reagent protocol (Invitrogen, Carlsbad, CA, USA). Total RNA was quantified using the NanoDrop® ND-2000 UV–VIS spectrophotometer (Thermo Scientific, Wilmington, DE, USA) at an OD of 260 nm, and the purity was assessed by determining the OD260/OD280 ratio. All of the samples had an OD260/OD280 ratio above 1.8 corresponding to 90-100% pure nucleic acids. Meanwhile, the integrity of RNA in each sample was assessed using 1% denatured agarose gel electrophoresis. RNA was used for quantitative RT-PCR analysis when the sample had a 28 S/18 S rRNA ratio ≥ 1.8 [30].

Total RNA was reverse transcribed using the PrimeScript® RT reagent kit with gDNA Eraser (Takara, Dalian, China) according to the manufacturer’s instruction. cDNA was synthesized and stored at −20°C until use. The RT-PCR analysis of gene expression was performed using primers for EGFR, AR, TNF-α, TLR4, and ribosomal protein L4 (RPL4) (Table 1), and the SYBR® Premix Ex Taq™ (Takara, Dalian, China) on an Applied Biosystems 7500 Real-Time PCR System (Foster City, CA, USA). The total volume of PCR reaction system was 50 μL. In brief, the reaction mixture contained 0.2 μM of each primer, 25 μL of SYBR® Premix Ex Taq™ (2×) and 4 μL of cDNA in a 50-μL reaction volume. All PCRs were done in triplicate on a 96-well RT-PCR plate under the following conditions (two-step amplification): 95°C for 30 sec, followed by 40 cycles of 95°C for 3 sec and 60°C for 30 sec. A subsequent melting curve (95°C for 15 sec, 60°C for 1 min and 95°C for 15 sec) with continuous fluorescence measurement and final cooling to room temperature was processed. Amplification products were verified by melting curves and agarose gel electrophoresis. Results were analyzed by the cycle threshold (C_T_) method [45]. Each biological sample was run in triplicate, RPL4 was used to standardize the relative expression of all genes investigated.

**Table 1:** Primers for RT-PCR analysis

**Gene**	**Primers**	
EGFR	Forward	5′- GGCCTCCATGCTTTTGAGAA -3′
	Reverse	5′- GACGCTATGTCCAGGCCAA -3′
AR	Forward	5′-GAGTACGATAACGAACCGCACA -3′
	Reverse	5′-TTTCCACTTTTGCCTCCCTTT -3′
TNF-α	Forward	5′- TCCAATGGCAGAGTGGGTATG -3′
	Reverse	5′- AGCTGGTTGTCTTTCAGCTTCAC -3′
TLR4	Forward	5′- GCCTTTCTCTCCTGCCTGAG -3′
	Reverse	5′- AGCTCCATGCATTGGTAACTAATG -3′
RPL4	Forward	5′- GAGAAACCGTCGCCGAAT -3′
	Reverse	5′- GCCCACCAGGAGCAAGTT -3′

The oligonucleotide primers were designed from pig gene sequences in the GenBank NM-2140075 (for EGFR), NM-214376 (for AR), NM-214022.1 (for TNF-α), AB188301 (for TLR4), and DQ845176 (for RPL4). To avoid amplification of potentially contaminating genomic DNA, the primers were designed to span introns and intron-exon boundaries.

### Statistical analysis

Data, expressed as means ± SD, were analyzed by one-way analysis of variance. The normality and constant variance for experimental data were tested by the Levene’s test. If data did not have homogenous variance, they underwent logarithm transformation to meet the necessary assumptions of analysis of variance [46]. Differences among treatment means were determined by Duncan’s multiple range tests. All statistical analyses were performed using SPSS 17.0 software (Chicago, IL, USA). *P* values < 0.05 were taken to indicate statistical significance [45].

## Results

### Growth performance

Average daily feed intake, average daily weight gain, and F/G (feed:gain) of the piglets between days 15 and 21 of the trial did not differ among the three groups (Table 2).

**Table 2:** Effects of NAC supplementation on the growth performance of AA-treated piglets (between days 15 and 21 of the trial)

**Items**	**Control**	**AA**	**NAC**
Average daily feed intake (g/day)	575 ± 3	576 ± 2	572 ± 4
Average daily weight gain (g/day)	301 ± 48	266 ± 69	267 ± 41
F/G (feed: gain)	1.9 ± 0.3	2.3 ± 0.7	2.2 ± 0.3

Data are means ± SD, n = 6. Control = piglets fed the basal diet and received intrarectal administration of sterile saline; AA = piglets fed the basal diet and received intrarectal administration of acetic acid; NAC = piglets fed the basal diet supplemented with 500 mg/kg NAC and received intrarectal administration of acetic acid.

### Intestinal morphometry

Colonic morphometric measurements are summarized in Table 3. The score for the AA group was significantly higher than the control and NAC group piglets (*P* < 0.05). AA administration caused a reduction (*P* < 0.05) in goblet cells/100 enterocytes and an increase (*P* < 0.05) in IEL/100 enterocytes and lymphocytic density. NAC supplementation increased (*P* < 0.05) goblet cells/100 enterocytes, decreased (*P* < 0.05) IEL/100 enterocytes and lymphocytic density, in comparison with the AA group.

**Table 3:** Effects of NAC supplementation on the colonic mucosal morphology of AA-treated piglets

**Items**	**Control**	**AA**	**NAC**
Score	6.3 ± 1.5^a^	12.5 ± 2.6^b^	8.3 ± 1.8^a^
Crypt Depth^1^, μm	190 ± 2.2	200 ± 3.5	231 ±3.8
Goblet cells/100 enterocytes	10.3 ± 2.2^b^	7.4 ±0.7^a^	9.8 ±1.6^b^
IEL^2^/100 enterocytes	1.6 ± 0.5^a^	2.7 ± 0.8^b^	1.8 ± 0.9^a^
Cell density^3^	1.3 ± 0.4	0.9 ± 0.3	1.0 ± 0.1
Lymphocytic density^3^	1.6 ± 0.2^a^	2.4 ± 0.7^b^	1.7 ± 0.3^a^

Data are means ± SD, n = 6. Control = piglets fed the basal diet and received intrarectal administration of sterile saline; AA = piglets fed the basal diet and received intrarectal administration of acetic acid; NAC = piglets fed the basal diet supplemented with 500 mg/kg NAC and received intrarectal administration of acetic acid. ^a-b:^ Values within a row with different letters differ (*P* < 0.05). ^1^Crypt depth: The distance from the crypt mouth to the base. The same crypt columns were used to determine the number of IEL, goblet cells expressed per 100 enterocytes. ^2^IEL = Intraepithelial lymphocytes. ^3^ Intravillus lamina propria cell and lymphocytic density expressed as number of total cells or number of lymphocytes per 1,000 μm^2^.

### Concentrations of DNA, RNA and protein in the colonic mucosa

Compared with the control group, AA treatment reduced protein/DNA ratio (*P <* 0.05) in the colonic mucosa (Table 4). Dietary NAC supplementation prevented such an effect of AA in piglets (*P <* 0.05). (Table 4).

**Table 4:** Effects of NAC supplementation on the colonic growth of AA-induced piglets

**Items**	**Control**	**AA**	**NAC**
DNA, mg/g	2.93 ± 0.19	3.20 ± 0.44	2.64 ± 0.38
RNA/DNA	4.55 ± 0.78	4.20 ± 1.44	4.45 ± 1.80
Protein/DNA	22.2 ± 3.8^b^	18.4 ± 1.6^a^	24.0 ± 3.2^b^
Protein/RNA	5.30 ± 0.64	4.55 ± 0.66	6.01 ± 1.92

Data are means ± SD, n = 6. Control = piglets fed the basal diet and received intrarectal administration of sterile saline; AA = piglets fed the basal diet and administered intrarectally with acetic acid; NAC = piglets fed the basal diet supplemented with 500 mg/kg NAC and received intrarectal administration of acetic acid. ^a-b:^ Values within a row with different letters differ (*P* < 0.05).

### Effects of NAC on redox status

Data on the redox status in plasma and colonic mucosa are illustrated in Table 5. Compared with the control, piglets in the AA group exhibited increases (*P* < 0.05) in the activities of MPO, the concentrations of MDA in the plasma and colon, as well as decreases (*P <* 0.05) in the activities of CAT in the colonic mucosa. In comparison with the AA piglets, NAC supplementation decreased (*P <* 0.05) the activities of MPO, and the concentrations of MDA in the plasma and colon.

**Table 5:** Effects of NAC on redox status in the plasma and colonic mucosa of AA-induced piglets

**Items**	**Control**	**AA**	**NAC**
Plasma			
MPO, U/ L	136 ± 12^a^	172 ± 24^b^	150 ± 24^a^
SOD, U/ mL	87.3 ± 30.2	82.0 ± 13.6	77.2 ± 11.8
CAT, U/ mL	4.58 ± 1.29	3.64 ± 1.03	6.50 ± 3.19
MDA, nmol/ mg protein	5.12 ± 0.51^a^	6.97 ± 1.24^b^	5.41 ± 1.02^a^
Colonic mucosa			
MPO, U/g wet mucosa	0.071 ± 0.003^a^	0.095 ± 0.018^b^	0.063 ± 0.016^a^
SOD, U/mg protein	20.9 ± 1.4	18.5 ± 4.2	20.5 ± 6.0
CAT, U/mg protein	1.16 ± 0.12^b^	0.99 ± 0.17^a^	0.87 ± 0.16^a^
MDA, nmol/mg protein	0.33 ± 0.04^a^	0.58 ± 0.16^b^	0.41 ± 0.15^a^

Data are means ± SD, n = 6. Control = piglets fed the basal diet and received intrarectal administration of sterile saline; AA = piglets fed the basal diet and received intrarectal administration of acetic acid; NAC = piglets fed the basal diet supplemented with 500 mg/kg NAC and received intrarectal administration of acetic acid. ^a-b:^ Values within a row with different letters differ (*P* < 0.05).

*MPO* Myeloperoxidase, *SOD* superoxide dismutase, *CAT* catalase, *MDA* malondialdehyde.

### Concentrations of inflammatory mediators in plasma and colonic mucosae, EGF in plasma and TGF-α in the colonic mucosa

Data on the concentrations of inflammatory mediators in plasma and colonic mucosae are shown in Table 6. Compared to the control, AA administration resulted in an increase (*P* < 0.05) in concentrations of TNF-α in plasma, PGE_2_ and TGF-α in the colonic mucosa. After treatment with NAC, the concentrations of TNF-α (*P* < 0.05) in plasma and of TGF-α (*P* < 0.05) in the colonic mucosa were decreased. Moreover, concentrations of EGF in the plasma of the NAC-supplemented piglets were increased (*P* < 0.05), compared with the AA group.

**Table 6:** Effects of NAC on proinflammatory mediators and growth modulator in the plasma and colonic mucosa

**Item**	**Control**	**AA**	**NAC**
Plasma			
TNF-α, ng/mL	0.61 ± 0.17^a^	0.84 ± 0.11^b^	0.49 ± 0.14^a^
IL-6, pg/mL	106.4 ± 23.6	115.2 ± 34.2	113.6 ± 18.3
PGE_2_, pg/mL	57.9 ± 11.5	55.1 ± 13.1	51.9 ± 11.8
EGF, ng/mL	0.65 ± 0.08^ab^	0.60 ± 0.07^a^	0.76 ± 0.10^b^
Colonic mucosa			
IL-6, pg/mL	134.3 ± 12.7	133.6 ± 17.2	130.8 ± 11.3
PGE_2_, pg/mL	74.5 ± 3.9^a^	96.0 ± 14.5^b^	90.5 ± 15.0^b^
TGF-α, pg/mL	3.50 ± 0.83^a^	4.28 ± 0.33^b^	2.56 ± 0.54^c^

Data are means ± SD, n = 6. Control = piglets fed the basal diet and received intrarectal administration of sterile saline; AA = piglets fed the basal diet and received intrarectal administration of acetic acid; NAC = piglets fed the basal diet supplemented with 500 mg/kg NAC and received intrarectal administration of acetic acid. ^a, b, c^ Values within a row with different letters differ (*P <* 0.05). *TNF-α* tumor necrosis factor-alpha, *IL-6* interleukin 6, *PGE*_*2*_ prostaglandin E_2_, *EGF* epidermal growth factor, *TGF-α* transforming growth factor-α.

### Abundance of caspase-3 and claudin-1 proteins in the colon mucosa

**Figure 1:**
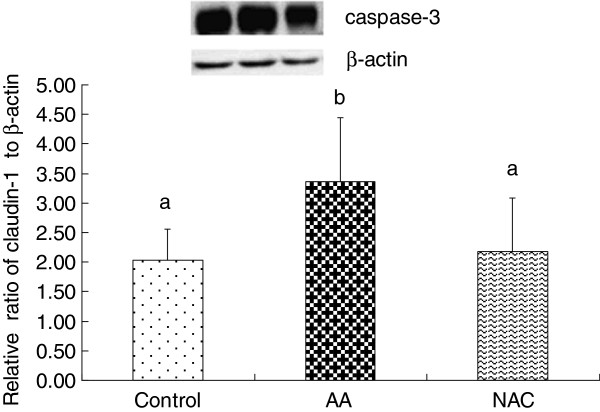
**Relative levels of caspase-3 expressed in the colonic mucosa of piglets.** Mucosal extracts (39 μg protein/sample) were separated by 10% SDS-polyacrylamide gel electrophoresis for determination of caspase-3 and β-actin. Values for relative caspase-3 abundance were normalized for β-actin. Data are means ± SD, n = 6. Control = piglets fed the basal diet and received intrarectal administration of sterile saline; AA = piglets fed the same control diet and received intrarectal administration of AA; NAC (AA + 500 mg/kg NAC) = piglets fed the basal diet supplemented with 500 mg/kg NAC and received intrarectal administration of AA. ^a, b^ Within the same intestinal segment, means with different superscripts differ (*P <* 0.05).

**Figure 2:**
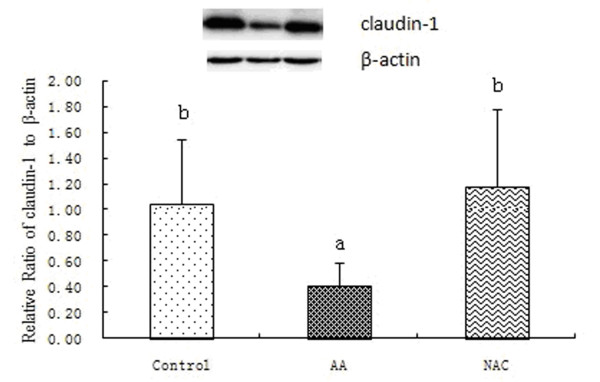
**Relative levels of claudin-1 expressed in the colonic mucosa of piglets.** Mucosal extracts (52 μg protein/sample) were separated by 12% SDS-polyacrylamide gel electrophoresis for determination of claudin-1 and β-actin. Values for relative claudin-1 abundance were normalized for β-actin. Data are means ± SD, n = 6. Control = piglets fed the basal diet and received intrarectal administration of sterile saline; AA = piglets fed the same control diet and received intrarectal administration of AA; NAC (AA + 500 mg/kg NAC) = piglets fed the basal diet supplemented with 500 mg/kg NAC and received intrarectal administration of AA. ^a, b^ Within the same intestinal segment, means with different superscripts differ (*P <* 0.05).

Caspase-3 and claudin-1 proteins were determined in the piglet colon (Figures 1 and 2). Compared with the control, AA administration caused an increase (*P <* 0.05) in the abundance of the caspase-3 protein and a decrease (*P <* 0.05) in the claudin-1 protein in the colonic mucosa. In contrast, NAC reduced (*P <* 0.05) the abundance of the caspase-3 protein and enhanced (*P <* 0.05) the abundance of the claudin-1 protein in the colonic mucosa.

### EGFR, AR, TNF-α and TLR4 mRNA levels in the colonic mucosa

Gene expression of EGFR, AR, TNF-α and TLR4 were measured in the colon mucosa by real-time PCR (Table 7). NAC supplementation markedly increased the abundance of EGFR mRNA (*P* < 0.05) compared to the control group. Piglets in the NAC group have much higher levels of AR mRNA (*P* < 0.05) than the other two groups. Also, colonic mucosal TNF-α mRNA levels were lower (*P* < 0.05) in the AA-treated piglets than in the control group and did not differ from that in the NAC group. TLR4 mRNA abundance did not differ among the three groups of pigs.

**Table 7:** Effects of NAC on EGFR, AR, TNF-α and TLR4 mRNA levels in the colonic mucosa

**Items**	**Control**	**AA**	**NAC**
EGFR	1.00 ± 0.29^b^	0.82 ± 0.19^ab^	0.61 ± 0.12^a^
AR	1.00 ± 0.17^a^	1.28 ± 0.20^a^	1.58 ± 0.17^b^
TNF-α	1.00 ± 0.16^b^	0.61 ± 0.16^a^	0.60 ± 0.11^a^
TLR4	1.00 ± 0.04	0.86 ± 0.34	0.71 ± 0.10

Data are means ± SD, n = 6. Control = piglets fed the basal diet and received intrarectal administration of sterile saline; AA = piglets fed the basal diet and received intrarectal administration of acetic acid; NAC = piglets fed the basal diet supplemented with 500 mg/kg NAC and received intrarectal administration of acetic acid. ^a-b:^ Values within a row with different letters differ (*P* < 0.05). EGFR = epidermal growth factor receptor; AR = amphiregulin; TNF-α = tumor necrosis factor-alpha; TLR4 = toll-like receptor 4.

## Discussion

**Figure 3:**
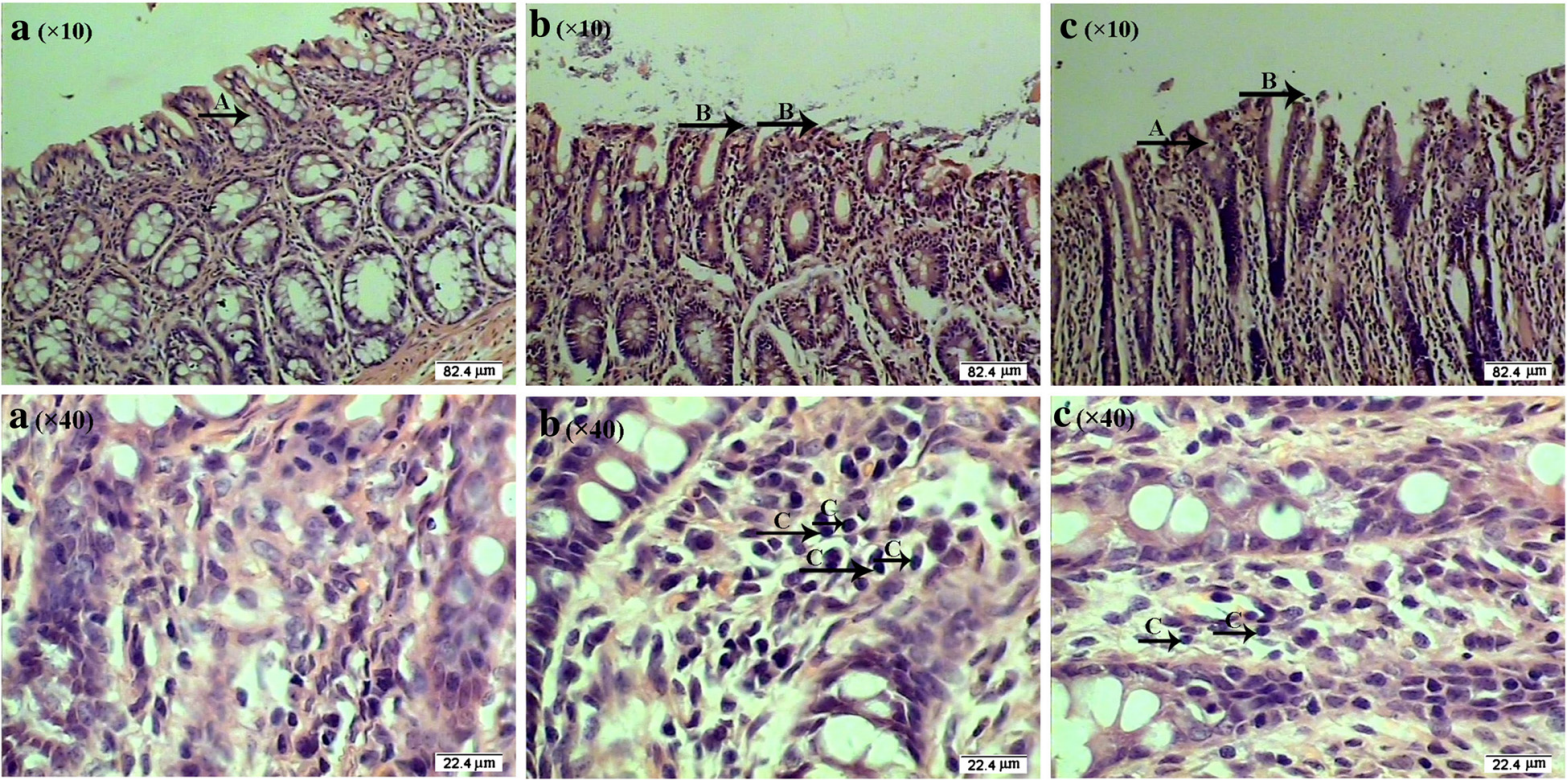
**Morphological change in the colon after intraluminal administration of acetic acid (AA). a**: control group; **b**: AA group, colon injury characterized by distortion of normal crypt architecture, loss of goblet cells, denuded epithelium, and infiltrating lymphocyte; **c**: NAC group, histological changes were significantly improved by NAC treatment. A: goblet cells; B: denuded epithelium; C: lymphocyte

**Figure 4:**
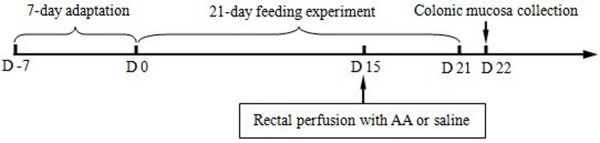
**The scheme of experimental design.** After a 7-day period of adaptation, eighteen piglets were assigned randomly into one of the three groups: control group, AA group, and NAC group. On day 15 of the trial, piglets in the AA and NAC groups received intrarectal administration of 10 mL of 10% AA, whereas piglets in the control group received the same volume of sterile saline. On day 22 of the trial, blood samples and colonic mucosae were collected.

The ulcerative colitis (UC) is a chronic inflammatory bowel disease with mucosal inflammation and ulceration of the colon [10]. In our study, dietary NAC supplementation could decrease gross mucosal injury caused by AA administration. The histopathology score for the AA group was significantly higher than that in the control and NAC groups (Table 3). The histologic ulcers showed necrosis in the colonic mucosa with submucosal inflammation, with neutrophils and lymphocytes being the predominant infiltrating cells. Moreover, we observed crypt abscesses, granulomatous inflammation with fibrosis, and massive thickening of the submucosa. Therefore, it could be concluded that the crypt of the colon in the AA group was elongated with distortion, while exhibiting the loss of epithelial cells, ulceration, lymphocyte infiltration, bowel wall thickening, and goblet cells depletion in the colon (Figure 3). These findings indicated that the UC model of piglet has been successfully developed in the present study. Fuss et al. [47] and Cetinkaya et al. [11] observed similar symptoms of macroscopic or microscopic colitis. Jensen et al. [48] also reported that colostrum increased mucin production by goblet cells as a first-line defense against bacterial attachment and invasion. Based on the biochemical parameters measured in the blood and colonic mucosa as well as data on growth performance, the experimental colitis at Day 7 post AA administration was relatively “mild”. In the present study, the AA administration increased the plasma and colon MPO activities by 1.26 and 1.34 times, respectively, and the plasma TNF-α level by 1.38 times, compared with the control group. In the rat model, AA-induced colitis was accompanied by an increase in MPO activity by 14.4 [11] or 5.36 times [49]. Similarly, MPO activity in the dextran sodium sulfate (DSS)-induced colitis model was 22 times [50] higher, and TNF-α level in plasma was approximately 100 times [51] higher than that for the control group. Because tissues were collected at Day 7 post AA administration in the present study (Figure 4), the period of 7 days was longer than that in other studies (e.g., 2 days or 5 days post administration of AA) [11,49], and we might have missed the time when stronger colitis occurred. In addition, to exclude a possible effect of AA-induced reduction in food intake on the piglet intestine, the control and NAC piglets were pair-fed the same amount of feed per kg body weight as AA piglets during days 15–21 of the trial (post-challenge with AA), which may eliminate an anorexic effect of AA treatment on young pigs. Nonetheless, we found that dietary supplementation with NAC increased the numbers of goblet cells and cell density, while decreaseing IEL and lymphocytic density. Furthermore, NAC prevented the AA-induced decrease in the protein/DNA ratio in the colon, which has been employed as an indicator of intestinal growth and development [52-54]. These results indicate that NAC could maintain the normal morphology of the colon and beneficially alleviate the AA-induced damage.

Emerging evidence suggests that members of the claudin-family of proteins play a critical role in tight junction formation and also affect the permeability characteristics in the gut [55]. Although the contribution of other tight junction proteins is less clear, up-regulation of claudin-1 appears to be a common mechanism by which colonic epithelial barrier function can be maintained and/or enhanced [56]. To extend these observations, we analyzed the relative level of claudin-1 expression in the colon mucosa. The results (Figure 2) showed that the abundance of claudin-1 protein in AA-induced piglets was decreased, when compared with the control group. Notably, dietary supplementation with NAC substantially increased the levels of claudin-1 in the colon mucosa, indicating that NAC may improve the colonic epithelial barrier function and alleviate the AA-induced mucosal damage in young pigs.

Ulcerative colitis is a chronic recurrent inflammatory bowel disease in which oxidative stress and cellular injury have been implicated [11,57]. This is consistent with elevated levels of TNF-α in the colonic mucosa of AA-treated piglets (Table 7). NAC appears to act primarily by increasing thiol antioxidant activity [58], thereby minimizing oxidative stress and the downstream negative effects of the stress [59]. MPO is an enzyme found predominantly in neutrophils and has been used as an effective quantitative index of inflammation due to a positive correlation between MPO activities and neutrophil infiltration in the colon [11,60]. MDA is an important indicator to reflect the extent of ROS accumulation in the body in response to oxidative damage [32]. Toxic colitic injury has been shown to increase MDA levels in rats [61,62]. Consistent with this report, we found that NAC supplementation decreased MPO in the plasma as well as MDA and TNF-α concentrations in the colon. These findings suggest that NAC could alleviate AA-induced oxidative injury in the colonic mucosa of piglets and may have positive effects on reducing the severity of colonic inflammation.

Oxidative stress and resultant tissue damage are the hallmark of cell death. Of particular note, NAC attenuated the production of active caspase-3 in the colon of AA-induced pigs (Figure 1). Apoptosis is typically accompanied by the activation of a class of "death" proteases (caspases) [63]. Caspase-3 stands out among the known caspases, because it is commonly activated by numerous "death" signals and cleaves a variety of important cellular proteins [64]. Thus, this protein is either partially or totally responsible for the proteolytic cleavage of many proteins. Our results demonstrated that NAC could effectively inhibit AA-induced apoptosis and promote cell growth and survival, indicating a protective effect of NAC against AA-induced colonocyte death through inhibiting the activation of caspase-3. These findings support the notion that NAC is effective in preventing intestinal oxidative injury and inflammatory disease in neonates [2].

Another novel and important observation of this study is an increase in EGF concentration in the plasma of NAC-supplemented pigs (Table 6). EGF can promote proliferation, repair, and migration of epithelial cells in the small intestine during the process of regeneration after its damage [30,65]. EGF can accelerate gastric ulcer healing by reducing bacterial colonization of the ulcer [66]. Epithelial mRNA levels for EGFR appears to be reduced or unchanged in patients with IBD [67]. In our porcine model of colitis, EGFR expression in the colon mucosa was not affected. Moreover, AR (a heparin-regulated growth factor) is a bifunctional growth modulator: it interacts with the EGF/TGF-α receptor to promote the growth of normal epithelial cells and inhibits the growth of certain aggressive carcinoma cell lines [68]. The AR's mRNA level is markedly elevated in the colon of NAC-supplemented piglets. AR could facilitate colonic injury recovery via its growth-regulatory effect.

## Conclusion

A piglet model of ulcerative colitis was successfully developed by intrarectal administration of 10 mL of 10% AA. This disorder was characterized by a deregulation of the colonic mucosal immune system along with the presence of architectural distortion and infiltration of neutrophils and macrophages. Dietary supplementation with 500 mg/kg NAC alleviated ulcerative colitic injury in AA-induced piglets. The beneficial effects of NAC were associated with the following changes: 1) alleviated colonic injury (indicated by a reduction in the AA-induced damage of the colonic structure), 2) reduced oxidative stress (indicated by decreased activities of MPO in the plasma, elevated levels of MDA in the plasma and colon), 3) reduced cell apoptosis (indicated by decreased expression of the caspase-3 protein in the colonic mucosa of AA-induced piglets), 4) enhanced recovery of the injured colon (increases in plasma EGF concentrations and colonic mucosal AR mRNA levels), and 5) enhanced formation of the tight junction (indicated by increased expression of claudin-1 proteins in the colonic mucosa of AA-induced piglets). Because AA produces colonic inflammation in rodents that resembles many histological characteristics of human ulcerative colitis [16], and because intestinal physiology and physiopathology are very similar between pigs and humans [69], our study helps to identify a beneficial role for dietary NAC supplementation as an adjuvant therapy for ulcerative colitis. Thus, findings from the porcine model may have important implications for the treatment of human intestinal disease (Crohn’s and ulcerative colitis).

## Abbreviations

AR: Amphiregulin; EGF: Epidermal growth factor; EGFR: Epidermal growth factor receptor; NAC: N-acetylcysteine; RPL4: Ribosomal protein L4; RT-PCR: Real-time polymerase-chain reaction; TLR4: Toll-like receptor 4; TNF-α: Tumor necrosis factor-alpha; TGF-α: Transforming growth factor-α.

## Competing interests

The authors declare that they have no competing interests.

## Authors’ contributions

YH and GW designed the study and wrote the manuscript. QW, DY, LW, BD, XC, and ML collected and analyzed experimental results. YL participated in the revision of the paper. All authors contributed to the data interpretation and approved the final version of the manuscript.

## Pre-publication history

The pre-publication history for this paper can be accessed here:

http://www.biomedcentral.com/1471-230X/13/133/prepub

## References

[B1] Wu G Fang YZ Yang S Lupton JR Turner ND Glutathione metabolism and its implications for health J Nutr 2004 134 489 492 14988435 10.1093/jn/134.3.489

[B2] Hou Y Wang L Zhang W Yang Z Ding B Zhu H Liu Y Qiu Y Yin Y Wu G Protective effects of N-acetylcysteine on intestinal functions of piglets challenged with lipopolysaccharide Amino Acids 2012 43 1233 1242 10.1007/s00726-011-1191-9 22180025

[B3] Wu G Amino acids: metabolism, functions, and nutrition Amino Acids 2009 37 1 17 19301095 10.1007/s00726-009-0269-0

[B4] Sridharan S Nalini R Shyamala DCS In vitro evaluation of the anticancer effect of N-acetylcysteine on oral carcinoma cell line Indian J Pharmacol 2001 33 343 349

[B5] Sadowska AM Manuel-y-Keenoy B De Backer WA Antioxidant and anti-inflammatory efficacy of NAC in the treatment of COPD: Discordant in vitro and in vivo dose-effects Pulm Pharmacol Ther 2007 20 9 22 10.1016/j.pupt.2005.12.007 16458553

[B6] Arakawa M Ito Y N-acetylcysteine and neurodegenerative diseases. Basic and clinical pharmacology Cerebellum 2007 6 308 314 10.1080/14734220601142878 17853088 PMC7102236

[B7] Dekhuijzen PNR Antioxidant properties of N -acetylcysteine: their relevance in relation to chronic obstructive pulmonary disease Eur Respir J 2004 23 629 636 10.1183/09031936.04.00016804 15083766

[B8] Cuzzocrea S Mazzon E Dugo L Serraino I Ciccolo A Centorrino T De Sarro A Caputi AP Protective effects of n-acetylcysteine on lung injury and red blood cell modification induced by carrageenan in the rat FASEB J 2001 15 1187 1200 10.1096/fj.00-0526hyp 11344087

[B9] Stadnicki A Colman RW Experimental models of inflammatory bowel disease Arch Immunol Ther Exp (Warsz) 2003 51 149 155 12894869

[B10] Kovvali G Das KM Molecular mimicry may contribute to pathogenesis of ulcerative colitis FEBS Lett 2005 579 2261 2266 10.1016/j.febslet.2005.02.073 15848155

[B11] Cetinkaya A Bulbuloglu E Kurutas EB Ciralik H Kantarceken B Buyukbese MA Beneficial effects of N-acetylcysteine on acetic acid-induced colitis in rats Tohoku J Exp Med 2005 206 131 139 10.1620/tjem.206.131 15888969

[B12] Jewell DP Patel C Immunology of inflammatory bowel disease Scand J Gastroenterol 1985 20 119 126 10.3109/00365528509093772 2935926

[B13] Keshavarzian A Morgan G Sedghi S Gordon JH Doria M Role of reactive oxygen metabolites in experimental colitis Gut 1990 31 786 790 10.1136/gut.31.7.786 2164491 PMC1378536

[B14] Millar AD Rampton DS Chander CL Claxson AW Blades S Coumbe A Panetta J Morris CJ Blake DR Evaluating the antioxidant potential of new treatments for inflammatory bowel disease using a rat model of colitis Gut 1996 39 407 415 10.1136/gut.39.3.407 8949646 PMC1383348

[B15] Jurjus AR Khoury NN Reimund JM Animal models of inflammatory bowel disease J Pharmacol Toxicolo Methods 2004 50 81 92 10.1016/j.vascn.2003.12.002 15385082

[B16] MacPherson BR Pfeiffer CJ Experimental production of diffuse colitis in rats Digestion 1978 17 135 150 10.1159/000198104 627326

[B17] Pawlik WW Hottensten OD Palen TE Pawlik T Jacobson ED Adenosine modulates reactive hyperemia in rat gut J Physiol Pharmacol 1993 44 119 137 8358049

[B18] Menguy R Desbaillets L Masters YF Mechanism of stress ulcer: Influence of hypovolemic shock on energy metabolism in the gastric mucosa Gastroenterology 1974 66 46 55 4809498

[B19] Wang L Walia B Evans J Gewirtz AT Merlin D Sitaraman SV IL-6 induces NF-κB activation in the intestinal epithelia J Immunol 2003 171 3194 3201 12960348 10.4049/jimmunol.171.6.3194

[B20] Feldmann M Brennan FM Maini RN Role of cytokines in rheumatoid arthritis Annu Rev Immunol 1996 14 397 440 10.1146/annurev.immunol.14.1.397 8717520

[B21] Polk WH Jr Dempsey PJ Russell WE Brown PI Beauchamp RD Barnard JA Coffey RJ Jr Increase production of transforming growth factor alpha following acute gastric injury Gastroenterol 1992 102 1467 1474 10.1016/0016-5085(92)91703-7 1568557

[B22] Konturek PC Ernst H Brzozowski T Ihlm A Hahn EG Konturek SJ Expression of epidermal growth factor and transforming growth factor alpha after exposure of rat gastric mucosa to stress Scand J Gastroenterol 1996 31 209 216 10.3109/00365529609004868 8833348

[B23] Blikslager AT Moeser AJ Gookin JL Jones SL Odle J Restoration of barrier function in injured intestinal mucosa Physiol Rev 2007 87 545 564 10.1152/physrev.00012.2006 17429041

[B24] Bergen WG Wu G Intestinal nitrogen recycling and utilization in health and disease J Nutr 2009 139 821 825 10.3945/jn.109.104497 19282369

[B25] Liu Y Huang J Hou Y Zhu H Zhao S Ding B Yin Y Yi G Shi J Fan W Dietary arginine supplementation alleviates intestinal mucosal disruption induced by Escherichia coli lipopolysaccharide in weaned pigs Br J Nutr 2008 100 552 560 10.1017/S0007114508911612 18275628

[B26] Hou Y Wang L Ding B Liu Y Zhu H Liu J Li Y Wu X Yin Y Wu G Dietary alpha-ketoglutarate supplementation ameliorates intestinal injury in lipopolysaccharide-challenged piglets Amino Acids 2010 39 555 564 10.1007/s00726-010-0473-y 20127262

[B27] Ademoglu E Erbil Y Tam B Barbaros U Ilhan E Olgac V Mutlu-Turkoglu U Do vitamin E and selenium have beneficial effects on trinitrobenzenesulfonic acid-induced experimental colitis Dig Dis Sci 2004 49 102 108 14992443 10.1023/b:ddas.0000011610.47179.0b

[B28] Yoshida N Yoshikawa T Yamaguchi T Naito Y Tanigawa T Murase H Kondo M A novel water-soluble vitamin E derivative protects against experimental colitis in rat Antioxid Redox Signal 1999 1 555 562 10.1089/ars.1999.1.4-555 11233152

[B29] Kuralay F Yildiz C Ozutemiz O Islekel H Caliskan S Bingol B Ozkal S Effects of trimetazidine on acetic acid-induced colitis in female swiss rats J Toxicol Environ Health A 2003 66 169 179 10.1080/15287390306402 12653021

[B30] Hou Y Wang L Yi D Ding B Yang Z Li J Chen X Qiu Y Wu G N-acetylcysteine reduces inflammation in the small intestine by regulating redox, EGF and TLR4 signaling Amino Acids 2013 45 513 522 10.1007/s00726-012-1295-x 22532030

[B31] Hou Y Yao K Wang L Ding B Fu D Liu Y Zhu H Liu J Li Y Kang P Yin Y Wu G Effects of α-ketoglutarate on energy status in the intestinal mucosa of weaned piglets chronically challenged with lipopolysaccharide Br J Nutr 2011 106 357 363 10.1017/S0007114511000249 21342606

[B32] Hou Y Wang L Ding BY Liu Y Zhu H Liu J Li Y Kang P Yin Y Wu G α-ketoglutarate and intestinal function Front Biosci 2011 16 1186 1196 10.2741/3783 21196226

[B33] Tan B Yin Y Liu Z Li X Xu H Kong X Huang R Tang W Shinzato I Smith SB Wu G Dietary L-arginine supplementation increases muscle gain and reduces body fat mass in growing-finishing pigs Amino Acids 2009 37 169 175 10.1007/s00726-008-0148-0 18683021

[B34] Nofrarías M Manzanilla EG Pujols J Gibert X Majó N Segalés J Gasa J Effects of spray-dried porcine plasma and plant extracts on intestinal morphology and on leukocyte cell subsets of weaned pigs J Anim Sci 2006 84 2735 2742 10.2527/jas.2005-414 16971575

[B35] Tan B Li XG Kong X Huang R Ruan Z Yao K Deng Z Xie M Shinzato I Yin Y Wu G Dietary L-arginine supplementation enhances the immune status in early-weaned piglets Amino Acids 2009 37 323 331 10.1007/s00726-008-0155-1 18712273

[B36] Li P Kim SW Li X Datta S Pond WG Wu G Dietary supplementation with cholesterol and docosahexaenoic acid affects concentrations of amino acids in tissues of young pigs Amino Acids 2009 37 709 716 10.1007/s00726-008-0196-5 18972185 PMC3716829

[B37] Wang J Chen L Li D Yin Y Wang X Li P Dangott LJ Hu W Wu G Intrauterine growth restriction affects the proteomes of the small intestine, liver and skeletal muscle in newborn pigs J Nutr 2008 138 60 66 18156405 10.1093/jn/138.1.60

[B38] Wang X Ou D Yin J Wu G Wang J Proteomic analysis reveals altered expression of proteins related to glutathione metabolism and apoptosis in the small intestine of zinc oxide-supplemented piglets Amino Acids 2009 37 209 218 10.1007/s00726-009-0242-y 19184341

[B39] Sartor RB Bond TM Schwab JH Systemic uptake and intestinal inflammatory effects of luminal bacterial cell wall polymers in rats with acute colonic injury Infect Immun 1988 8 2101 2108 3397186 10.1128/iai.56.8.2101-2108.1988 PMC259529

[B40] Heller F Fuss IJ Nieuwenhuis EE Blumberg RS Strober W Oxazolone colitis, a Th2 colitis model resembling ulcerative colitis, is mediated by IL-13-producing NK-T cells Immunity 2002 17 629 638 10.1016/S1074-7613(02)00453-3 12433369

[B41] Medina C Videla S Radomski A Radomski M Antolín M Guarner F Vilaseca J Salas A Malagelada JR Therapeutic effect of phenantroline in two rat models of inflammatory bowel disease Scand J Gastroenterol 2001 36 1314 1319 10.1080/003655201317097182 11761023

[B42] Prasad AS DeMouchelle E Koniuchi D A simple fluorimetric method for the determination of RNA and DNA in tissue J Lab Clin Med 1972 80 598 601 5073899

[B43] Munro HN Fleck A Munro HN Analysis of tissues and body fluids for nitrogenous constituents Mammalian protein metabolism 1969 New York: Academic press 465 483

[B44] Lowry OH Rosebrough NJ Farr AL Protein measurement with the folin phenol reagent J Biol Chem 1951 193 265 275 14907713

[B45] Fu WJ Stromberg AJ Viele K Carroll RJ Wu G Statistics and bioinformatics in nutritional sciences: analysis of complex data in the era of systems biology J Nutr Biochem 2010 21 561 572 10.1016/j.jnutbio.2009.11.007 20233650 PMC2885517

[B46] Wei JW Carroll RJ Harden KK Wu G Comparisons of treatment means when factors do not interact in two-factorial studies Amino Acids 2012 42 2031 2035 10.1007/s00726-011-0924-0 21547361 PMC3199378

[B47] Fuss IJ Boirivant M Lacy B Strober W The interrelated roles of TGF- and IL-10 in the regulation of experimental colitis J Immunol 2002 168 900 908 11777988 10.4049/jimmunol.168.2.900

[B48] Jensen M Puiman P Stoll B Dorst K Renes I Sangild P Van GoudoeverJ: **Improved gut barrier function via increased threonine utilization may explain enhanced resistance to necrotizing enterocolitis in preterm pigs fed colostrum** Acta Paediatr 2009 98 44

[B49] Choudhary S Keshavarzian A Yong S Wade M Bocckino S Day BJ Banan A Novel antioxidants zolimid and aeol11202 ameliorate colitis in rats Dig Dis Sci 2001 46 2222 2230 10.1023/A:1011975218006 11680601

[B50] Laroui H Ingersoll SA Liu HC Baker MT Ayyadurai S Charania MA Laroui F Yan Y Sitaraman S Merlin D Dextran sodium sulfate (DSS) induces colitis in mice by forming nano-lipocomplexes with medium-chain-length fatty acids in the colon PLoS ONE 2012 7 e32084 10.1371/journal.pone.0032084 22427817 PMC3302894

[B51] Alex P Zachos NC Nguyen T Gonzales L Chen TC Conklin LS Centola M Li XH Distinct cytokine parttens identified from multiplex profiles of murine DSS and TNBS-induced colitis Inflmm Bowel Dis 2009 15 341 352 10.1002/ibd.20753 PMC2643312 18942757

[B52] Fasina YO Moran ET Ashwell CM Conner DE Leslie M Mckee SR Effect of dietary gelatin supplementation on the expression of selected enterocyte genes, intestinal development and early chick performance Int J Poultry Sci 2007 6 944 951 10.3923/ijps.2007.944.951

[B53] Iji PA Saki A Tivey DR Intestinal development and body growth of broiler chicks on diets supplemented with non-starch polysaccharides Anim Feed Sci Technol 2001 89 175 188 10.1016/S0377-8401(00)00223-6

[B54] Jeurissen SH Lewis F van der Klis JD Mroz Z Rebel JM ter Huurne AA Parameters and techniques to determine intestinal health of poultry as constituted by immunity, integrity and functionality Curr Issues Intest Microbiol 2002 3 1 14 12022808

[B55] Tsukita S Furuse M The structure and function of claudins, cell adhesion molecules at tight junctions Ann NY Acad Sci 2000 915 129 135 11193568 10.1111/j.1749-6632.2000.tb05235.x

[B56] Howe KL Reardon C Wang A Nazli A McKay DM Transforming growth factor-1 regulation of epithelial tight junction proteins enhances barrier function and blocks enterohemorrhagic Escherichia coli O157:H7-induced increased permeability Am J Pathol 2005 167 1587 1597 10.1016/S0002-9440(10)61243-6 16314472 PMC1613202

[B57] Seril DN Liao J Yang GY Yang CS Oxidative stress and ulcerative colitis-associated carcinogenesis: studies in humans and animal models Carcinogenesis 2003 24 353 362 10.1093/carcin/24.3.353 12663492

[B58] Gürer H Ozgünes H Neal R Spitz DR Erçal N Antioxidant effects of N-acetylcysteine and succimer in red blood cells from lead-exposed rat Toxicology 1998 128 181 189 10.1016/S0300-483X(98)00074-2 9750041

[B59] Dodd S Dean O Copolov DL Malhi GS Berk M N-acetylcysteine for antioxidant therapy: pharmacology and clinical utility Expert Opin Biol Ther 2008 8 1955 1962 10.1517/14728220802517901 18990082

[B60] Krawisz JE Sharon P Stenson WF Quantitative assay for acute intestinal inflammation based on myeloper-oxidase activity Gastroenterology 1984 87 1344 1350 6092199

[B61] Liu SP Dong WG Wu DF Luo HS Yu JP Protective effect of angelica sinensis polysaccharide on experimental immunological colon injury in rats World J Gastroenterol 2003 9 1790 2786 10.3748/wjg.v9.i12.2786 PMC4612053 14669334

[B62] Mahgoub AA El-Medany AA Hager HH Mustafa AA El-Sabah DM Evaluating the prophylactic potential of zafirlukast against the toxic effects of acetic acid on the rat colon Toxicol Lett 2003 145 79 87 10.1016/S0378-4274(03)00269-8 12962976

[B63] Nicholson DW Thornberry NA Caspases: killer proteases Trends Biochem Sci 1997 22 299 306 10.1016/S0968-0004(97)01085-2 9270303

[B64] Jänicke RU Sprengart ML Wati MR Porter AG Caspase-3 is required for DNA fragmentation and morphological changes associated with apoptosis J Biol Chem 1998 273 9357 9360 10.1074/jbc.273.16.9357 9545256

[B65] Nair RR Warner BB Warner BW Role of epidermal growth factor and other growth factors in the prevention of necrotizing enterocolitis Semin Perinatol 2008 32 107 113 10.1053/j.semperi.2008.01.007 18346534

[B66] Elliott SN Wallace JL McKnight W Gall DG Hardin JA Olson M Buret A Bacterial colonization and healing of gastric ulcers: the effects of epidermal growth factor Am J Physiol Gastrointest Liver Physiol 2000 278 G105 G112 10644568 10.1152/ajpgi.2000.278.1.G105

[B67] Chowdhury A Fukuda R Fukumoto S Growth factor mRNA expression in normal colorectal mucosa and in uninvolved mucosa from ulcerative colitis patients J Gastroenterol 1996 31 353 360 10.1007/BF02355024 8726826

[B68] Plowman GD Green JM McDonald VL Neubauer MG Disteche CM Todaro GJ Shoyab M The amphiregulin gene encodes a novel epidermal growth factor-related protein with tumor-inhibitory activity Mol Cell Biol 1990 10 1969 1981 2325643 10.1128/mcb.10.5.1969-1981.1990 PMC360543

[B69] Sodhi C Richardson W Gribar S Hackam DJ The development of animal models for the study of necrotizing enterocolitis Dis Model Mech 2008 1 94 98 10.1242/dmm.000315 19048070 PMC2562191

